# Peptide SS-31 upregulates frataxin expression and improves the quality of mitochondria: implications in the treatment of Friedreich ataxia

**DOI:** 10.1038/s41598-017-10320-2

**Published:** 2017-08-29

**Authors:** Hongting Zhao, Huihui Li, Shuangying Hao, Jiping Chen, Jing Wu, Chuanhui Song, Meng Zhang, Tong Qiao, Kuanyu Li

**Affiliations:** 1 Jiangsu Key Laboratory of Molecular Medicine, Medical School of Nanjing University, 210093 Nanjing, P. R. China; 2 Department of Vascular Surgery, Drum Tower Clinical Medical College of Nanjing Medical University, Nanjing, P. R. China; 3 Present Address: Medical School of Henan Polytechnic University, 454000 Jiaozuo, P. R. China

## Abstract

Friedreich ataxia is a progressive neurodegenerative disease caused by the expansion of GAA trinucleotide repeats within the first intron of the *FXN* gene, which encodes frataxin. The pathophysiology of the disease is thought to be derived from the decrease of Fe-S cluster biogenesis due to frataxin deficiency. There is currently no effective treatment for the disease. In our study, we demonstrated that treatment with the mitochondrion-targeted peptide SS-31 reduced frataxin deficiency-induced oxidative stress in lymphoblasts and fibroblasts derived from patients. Interestingly, SS-31 treatment translationally upregulated the protein level of frataxin in a dose-dependent manner. Furthermore, SS-31 treatment increased the enzymatic activities of the iron-sulphur enzymes, including aconitase and complex II and III of the respiratory chain. Further evaluation of the quality of mitochondria showed that mitochondrial membrane potential, ATP content, NAD^+^/NADH, and the morphology of mitochondria all improved. Our results suggest that SS-31 might potentially be a new drug for the early treatment of Friedreich ataxia.

## Introduction

Friedreich ataxia (FRDA, Online Mendelian Inheritance in Man database #229300) is a severe neurodegenerative disease that is the most common recessive ataxia in the Caucasian population^1^. FRDA, caused by decreased expression of frataxin (FXN), is characterised by a mixed spinocerebellar and sensory ataxia frequently associated with cardiomyopathy^2^. The decrease of FXN stems from an expansion of GAA triplet repeats in the first intron of the gene *FXN*. This GAA expansion leads to transcriptional silencing of *FXN* through a mechanism involving modifications of the chromatin structure at the locus, resulting in the expression of a structurally and functionally normal FXN but at levels that are estimated to be 5–30% of normal levels^3^. FXN is important in iron-sulphur cluster assembly, where it likely functions as a chaperone that provides iron in a bioavailable form in the early steps of iron–sulphur cluster biosynthesis^4^, or as an allosteric factor that modulates the cysteine desulfurase activity^5–7^. Patients manifest mitochondrial iron–sulphur cluster assembly deficiency with decreased activities of iron–sulphur proteins such as mitochondrial aconitase and complex I, II, and III^8^. Moreover, the role of FXN in preventing the formation of deleterious reactive oxygen species (ROS) has been well established^9^, invoking an additional paradigm of FRDA pathology, in which ROS toxicity leads to mitochondrial dysfunction with subsequent neuron death.

No cure or effective treatment for FRDA has yet been reported, though a few clinical trials have made some limited progress^10^. MitoQ and idebenone are antioxidants and serve to eliminate free radicals. Preliminary testing revealed idebenone to be a safe treatment for FRDA, exhibiting a positive effect on cardiac hypertrophy and neurological function^11–14^. However, the drug was not approved for FRDA due to the failure of the drug to show efficacy in further clinical trials^15^. No available data for MitoQ in a clinical trial for FRDA is available. Another therapeutic approach for FRDA is epigenetic modulation through heterochromatin acetylation by histone deacetylase inhibitor (HDACi) to increase the mRNA and protein levels of FXN^16, 17^. Unfortunately, the final results showed no improvement over placebo in patients in phase III trials. Due to iron accumulation in mitochondria when FXN is deficient, treatment by selective iron chelation was proposed for FRDA^18, 19^. A combined therapy of a low dose of deferiprone, an iron chelator, with idebenone was conducted, which likely improved neurological function and heart hypertrophy^20^. For long term use, this might not be a suitable choice because iron deprivation can downregulate FXN expression^21^. At present, gene therapy in animal and cell models showed some beneficial effect^22, 23^. More recently, researchers used synthetic DNA or RNA to block R-loop formation, thereby triggering *FXN* gene activation to levels similar to analogous wild-type cells^24^. This creates a new candidate strategy for FRDA treatment.

Here, we report a potential therapeutic strategy for FRDA with a tetra-peptide, Szeto-Schiller (SS)-31. SS-31 is a novel mitochondrion-targeted antioxidant, which accumulates in the inner mitochondrial membrane^25^, where the electron transport chain takes place and ROS is produced as a by-product. SS-31 interacts with mitochondrial cardiolipin to maintain the mitochondrial structure and integrity. Previous studies have shown that SS-31 treatment improves ATP production, reduces mitochondrial ROS production, and decreases oxidative damage. SS-31 has been demonstrated to be highly effective in several animal models associated with mitochondrial dysfunction and oxidative stress, such as ischemia-reperfusion injury and heart failure^26–28^.

In our study, we examined the effect of SS-31 treatment on lymphoblasts and fibroblasts derived from FRDA patients and found that SS-31 not only acts as an antioxidant to relieve the oxidative stress, but also increases the protein level of FXN, which would be the fundamental resolution for FRDA patients. Therefore, the aim of our study is to investigate whether the upregulated FXN expression induced by SS-31 treatment can improve mitochondrial function.

## Results

### SS-31 treatment upregulates FXN expression in FRDA patient-derived cells

**Figure 1:**
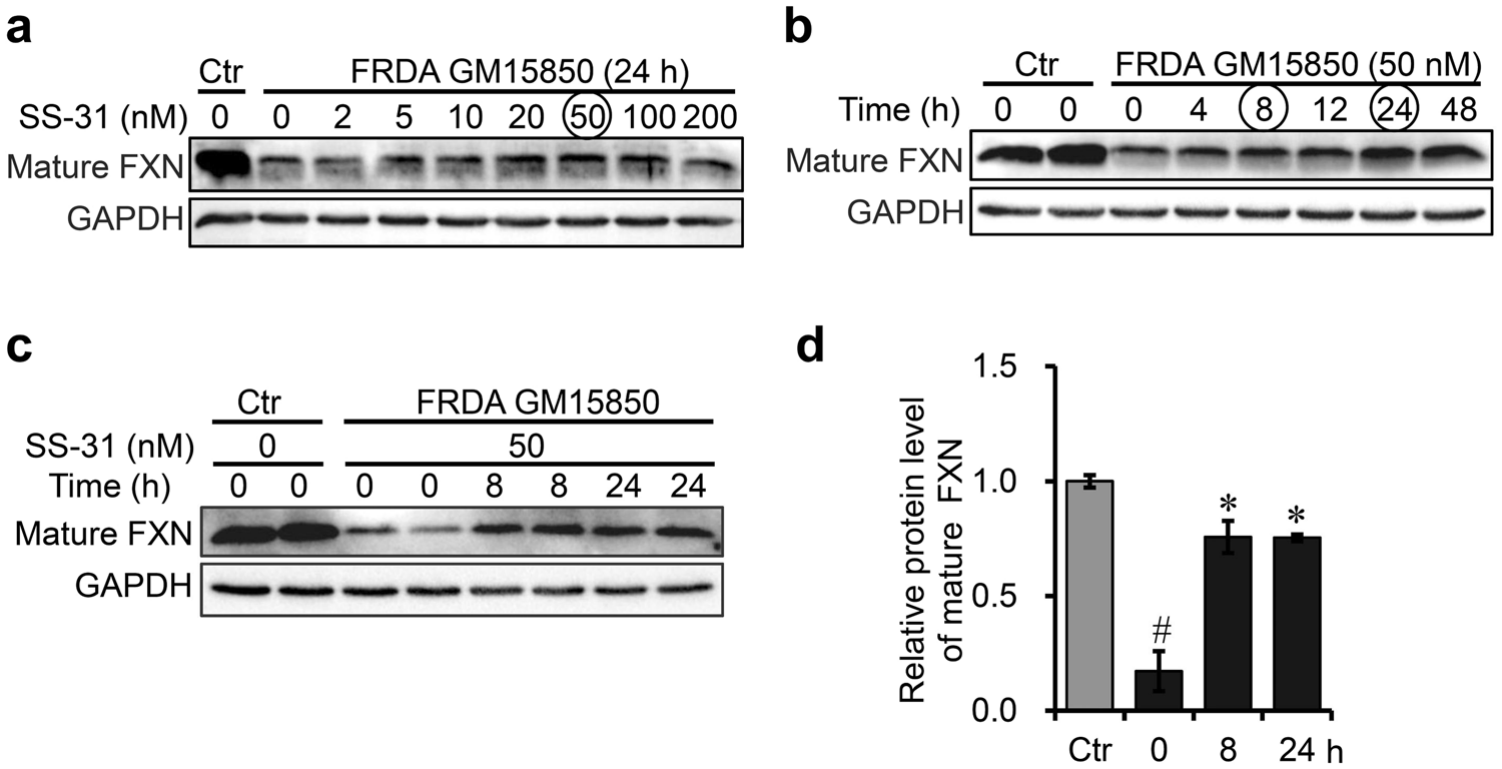
FXN expression is upregulated by SS-31 treatment in GM15850 Cells. (**a,b**) Western Blotting analysis to optimise the condition, under which SS-31 treatment induced the expression of FXN in lymphoblasts (GM15850) derived from a FRDA patient. Ctr: GM15849 derived from a matched healthy subject. The patient-derived lymphoblasts were treated with SS-31 in different concentrations and time points. (**c**) A representative image of Western blot to show the protein level of FXN in patient-derived lymphoblasts after treatment with 50 nM of SS-31 for 8 or 24 h. (**d**) Quantification of the FXN protein level after SS-31 treatment in lymphoblasts derived from a patient reaching 75% of healthy control. Values represent mean ± SEM (n = 3, each duplicates). A one-way ANOVA was carried out, ^#^ *p < *0.05 compared with Ctr; *p < 0.05 compared with the untreated cells (t = 0). GAPDH was used as an internal control.

Efforts to find a drug capable of increasing the ability to cope with oxidative stress have long been made in the treatment of FRDA. Based on the characteristics of SS-31, a mitochondrion-targeted antioxidant, we chose it as a potential drug. Very strikingly, we found that SS-31 treatment increased the expression of FXN (Fig. 1). This prompted us to evaluate the value of the peptide. First, we optimised the condition for inducing the expression of FXN and found that the protein level of FXN increased in a dose dependent manner in both lymphoblasts and fibroblasts derived from patients (Fig. 1a and Fig. S1). Relatively, FXN expression was much more robust in lymphoblasts than in fibroblasts derived from healthy controls (unpublished data) and the response to SS-31 treatment was stronger in lymphoblasts than in fibroblasts derived from patients (Fig. 1a and Fig. S1). Thus, lymphocytes (GM15850) from FRDA patient are more straightforward for further study. The optimal concentration of SS-31 was determined to be within the range of 20 to 50 nM (Fig. 1a). A time course of the treatment was assessed, and the highest level was reached within 24 h (Fig. 1b). Finally, we chose 50 nM and 8 and 24 h as optimal conditions for later experiments.

Interestingly, our data showed that SS-31 treatment had no effect on FXN expression in healthy cells (Fig. S2), but significantly increased the protein level of FXN in cells derived from FRDA patients to reach 75% of the level of healthy control-derived cells (designated as “healthy control” later on) (Fig. 1d). To see if the effect of the upregulation stemmed from its antioxidant function, we used two common antioxidants lipoic acid (LA) and ascorbic acid (AA) as controls to check if FXN expression could be upregulated by them. Neither LA nor AA treatment had any effect on the protein level of FXN in patient-derived cells (Fig. S3). Thus, the effect of SS-31 on the upregulation of FXN is a novel function, and it is rational to further characterise the effect of SS-31-induced upregulation of FXN on iron metabolism and mitochondrial function.

### SS-31 treatment improves the quality of mitochondria in FRDA patient-derived cells

**Figure 2:**
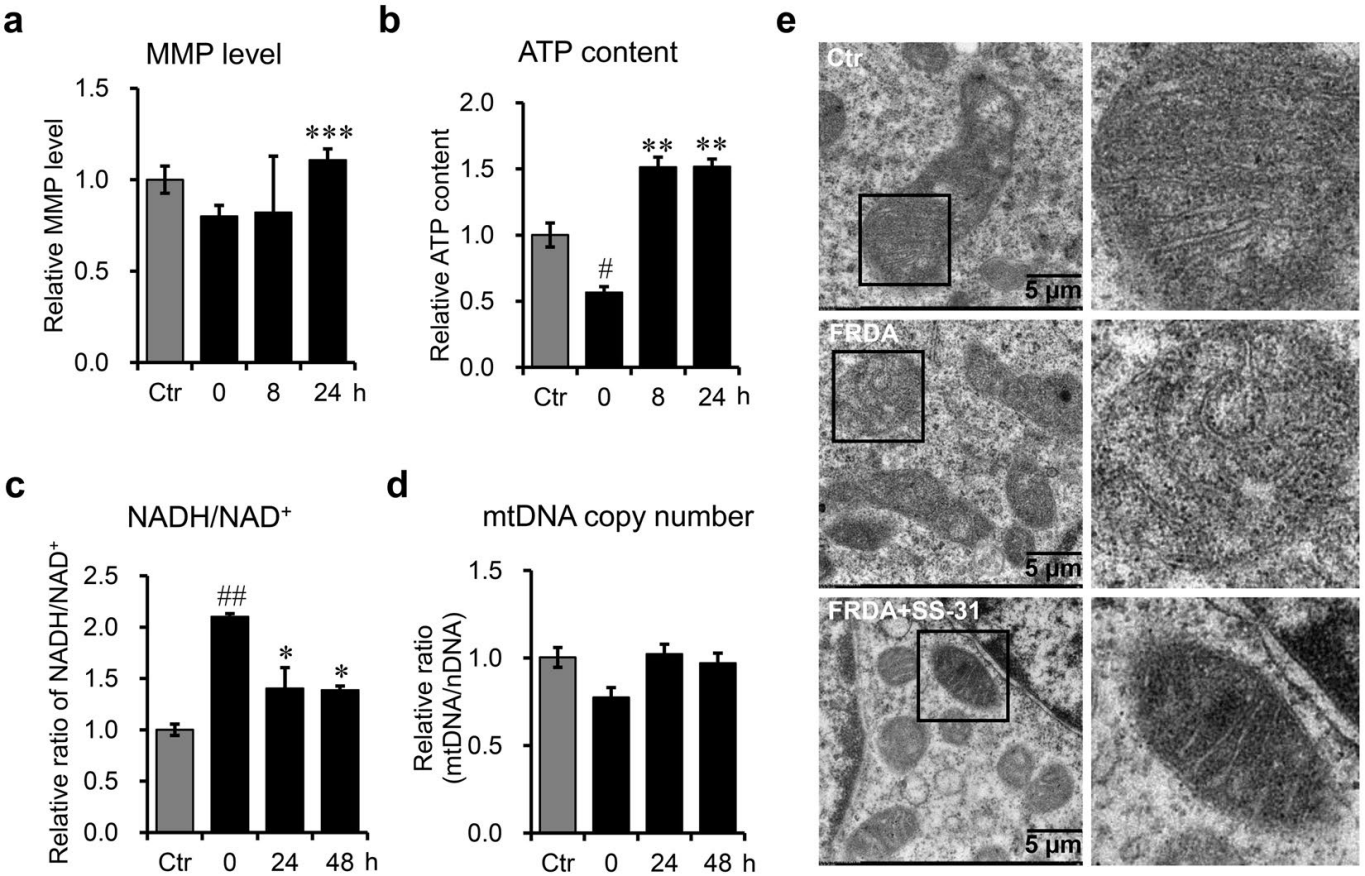
SS-31 improves the quality and quantity of mitochondria in GM15850 cells after SS-31 treatment. (**a**) Mitochondrial membrane potential (MMP), examined with a fluorescent probe JC-1. (**b**) ATP content. (**c**) Ratio of NADH/NAD^+^. (**d**) Relative copy number of mitochondrial DNA (mtDNA) normalised to nuclear DNA (nDNA). *CytB* represents mtDNA genome; *β-actin* represents nuclear genome. Values represent mean ± SEM (n = 4, each duplicates). (**e**) Mitochondrial morphology revealed by electron transmission microscopy. Ctr: GM15849; FRDA: GM15850; FRDA+SS-31: GM15850 cells treated with SS-31 for 24 h. Values represent mean ± SEM (n = 3, each duplicates). A one-way ANOVA was carried out, ^#^ *p* < 0.05, compared with Ctr. *p < 0.05, **p < 0.01, ***p < 0.001, compared with the untreated GM15850 cells.

FRDA is a mitochondrial disease. Mitochondria derived from FRDA patient cells exhibit structural and functional impairment. Mitochondrial membrane potential (MMP) is considered as an indicator of mitochondrial integrity and bioenergetic function. To investigate the effects of SS-31 on mitochondria in cells derived from FRDA patients, we detected MMP with the lipophilic dye JC-1 by measuring a potential-dependent shift in fluorescence from green to red, which reflected its aggregation in mitochondria^29^. The increased ratio of red versus green fluorescence in patient-derived cells after SS-31 treatment indicated more polarised mitochondria (Fig. 2a). Intracellular ATP level is another pivotal measure of mitochondrial quality. We found that SS-31 treatment significantly raised ATP levels in patient-derived cells (Fig. 2b), indicating increased oxidative phosphorylation. The ratio of NADH/NAD^+^ in patient-derived lymphoblasts was also measured and was found to be significantly reduced to levels comparable to the healthy control cells (Fig. 2c). These results indicate much improvement of mitochondrial quality in patient-derived lymphoblasts post SS-31 treatment. Furthermore, we quantified the copy number of mitochondrial DNA and found that SS-31 treatment mildly increased the copy number of mitochondria in patient-derived lymphoblasts (Fig. 2d). Electron microscopic data substantiated these results, revealing structural improvements from abnormal cristae in patient-derived cells to regular invagination of the inner membrane after SS-31 treatment (Fig. 2e). Taken together, SS-31 improved mitochondrial quality significantly and increased mitochondria quantity mildly in patient-derived cells.

### SS-31 treatment enhances the activities of mitochondrial iron-sulphur cluster-containing enzymes

**Figure 3:**
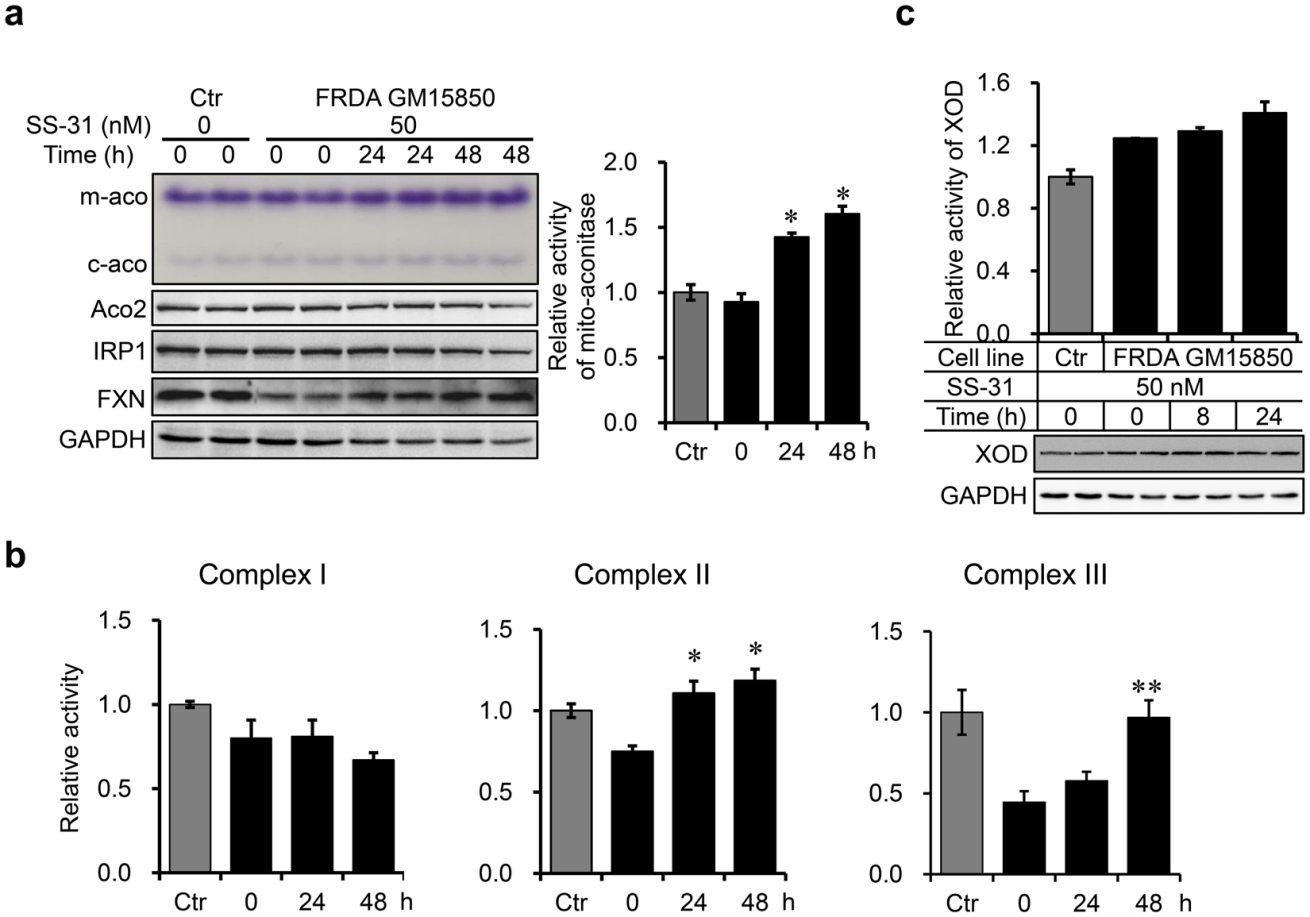
SS-31 treatment increases the enzymatic activities of mitochondrial Fe-S enzymes. (**a**) Mitochondrial (m-aco) and cytosolic (c-aco) aconitase activities revealed by in-gel assays (left upper panel) and quantified (right panel). m-aco activity significantly increased after SS-31 treatment, but c-aco activity only mildly in FRDA patient-derived cells (GM15850). Related protein levels are shown below the aconitase activity panel. Ctr: GM15849. (**b**) The activities of complex I, II, and III after SS-31 treatment in FRDA patient derived cells. (**c**) XOD activity (bar panel) and protein levels (revealed by western blotting). Values represent mean ± SEM (n = 3, each duplicates). A one-way ANOVA was carried out, *p < 0.05, **p < 0.01 compared with the untreated cells derived from the FRDA patient.

A deficiency in iron-sulphur cluster biogenesis is considered as the primary outcome of FXN deficiency. To check whether the increased FXN protein level was sufficient to improve iron-sulphur cluster biogenesis, we measured the activities of a number of iron-sulphur cluster–containing enzymes. First, in-gel activity assays showed that the mitochondrial aconitase activity (m-aco) in FRDA patient-derived cells increased significantly after SS-31 treatment. The cytosolic aconitase activity (c-aco) increased as well, but mildly (Fig. 3a). However, the protein levels of the corresponding enzymes did not change (Fig. 3a, ACO2 and IRP1). Complex I, II, and III of the respiratory chain are the most abundant iron-sulphur cluster-containing enzymes in mitochondria. The activities of these enzymes in FRDA patient-derived cells are all reduced due to insufficient biogenesis of iron-sulphur clusters. No improvement in complex I was observed, while SS-31 treatment improved the activities of complex II and complex III (Fig. 3b). Xanthine oxidoreductase (XOD), an enzyme that generates ROS, needs iron-sulphur cluster as a prosthetic group. We found that the activity of XOD showed no changes after SS-31 treatment (Fig. 3c). These results suggest that SS-31-induced FXN upregulation facilitates iron-sulphur cluster biogenesis in the mitochondria to specifically promote the activities of several iron-sulphur cluster-containing enzymes.

### SS-31 treatment modulates the iron metabolism of cells derived from FRDA patients

**Figure 4:**
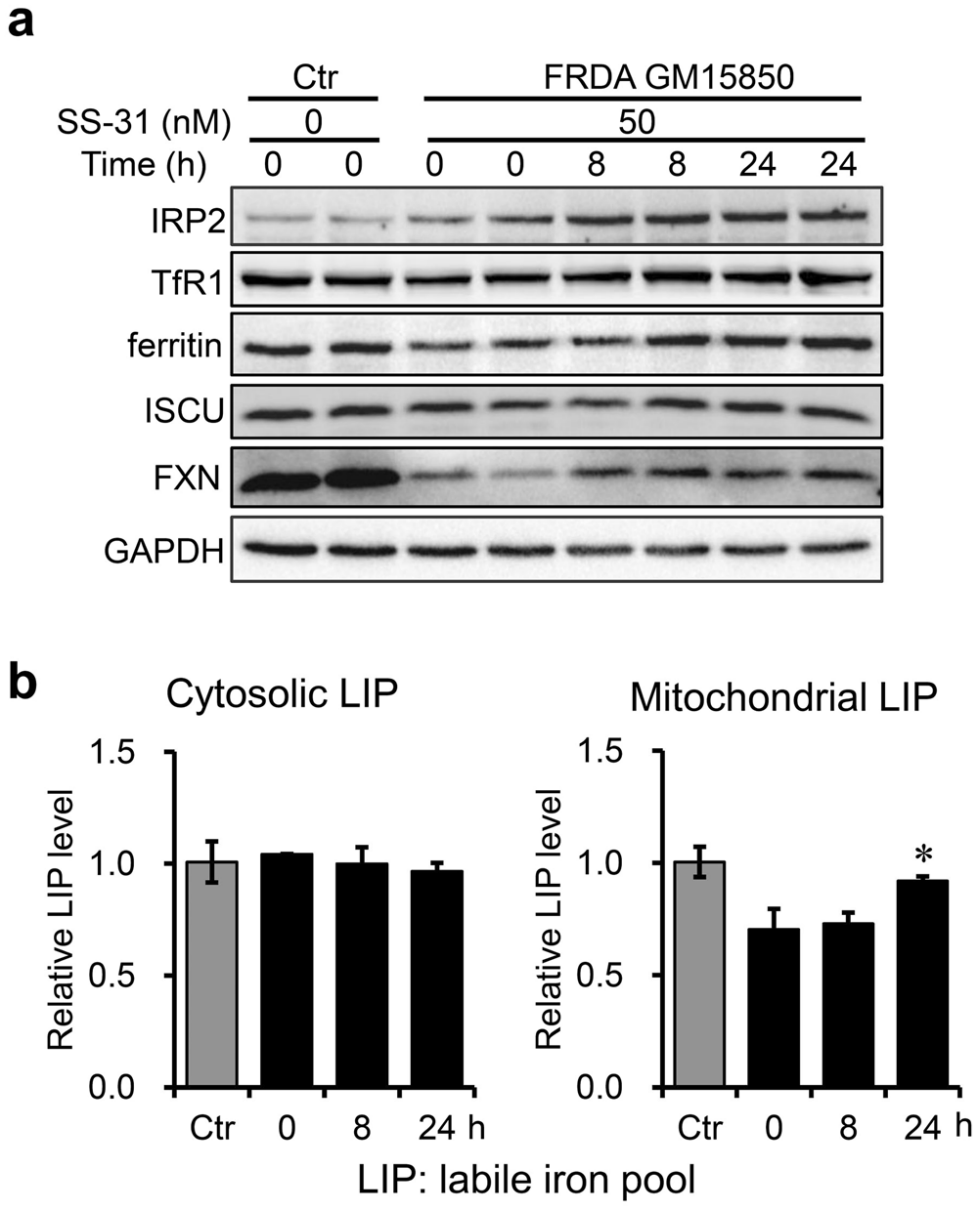
SS-31 treatment modulates the iron metabolism of cells derived from a FRDA patient. (**a**) Effects of SS-31 treatment on the expression of iron-related proteins in GM15850 cells. The cells were treated with 50 nM of SS-31 for 8 or 24 h. Total protein was collected and lysates were probed by western blotting analysis for the expression of iron-related proteins (IRP2, TfR1, ferritin, ISCU, FXN). GAPDH was used as an internal control. (**b**) Relative cytosolic and mitochondrial LIP after SS-31 treatment in GM15850 cells. The cells were treated with 50 nM of SS-31 for 8 and 24 h and stained with 10 µM Calcein-AM or 10 µM RPA. The cytosolic and mitochondrial LIP were determined by the fluorescence intensity (see Materials and Methods). Values represent mean ± SEM (n = 3, each duplicates). A one-way ANOVA was carried out, *p < 0.05 compared with the untreated cells.

To study the effects of SS-31 on the iron metabolism in FRDA patient-derived cells, we evaluated the expression of iron related proteins and measured the levels of cytosolic and mitochondrial labile iron pool (LIP). The expression level of ferritin in patient-derived cells increased to a level comparable to healthy control 24 h post SS-31 treatment, indicating that iron uptake was enhanced. In agreement with the result, both protein levels of IRP2 and TfR1 after SS-31 treatment increased, although IRP2 protein levels were already higher in patient-derived cells than in healthy control^21^ (Fig. 4a). Then we used the fluorescent probe Calcein-AM to evaluate cytosolic LIP and RPA (rhodamine B-[(1,10-phenanthrolin-5-yl) aminocarbonyl] benzylester) for mitochondrial LIP. Cytosolic LIP level remained constant, in contrast to the increased level of ferritin (Fig. 4a and b). However, mitochondrial LIP level was significantly elevated 24 h post SS-31 treatment (Fig. 4b, right panel), indicating an increase in bioavailable iron in mitochondria. These results demonstrate that SS-31 treatment modulates cellular iron metabolism.

### SS-31 treatment decreases ROS level and enhances the antioxidative ability of FRDA patient-derived cells

**Figure 5:**
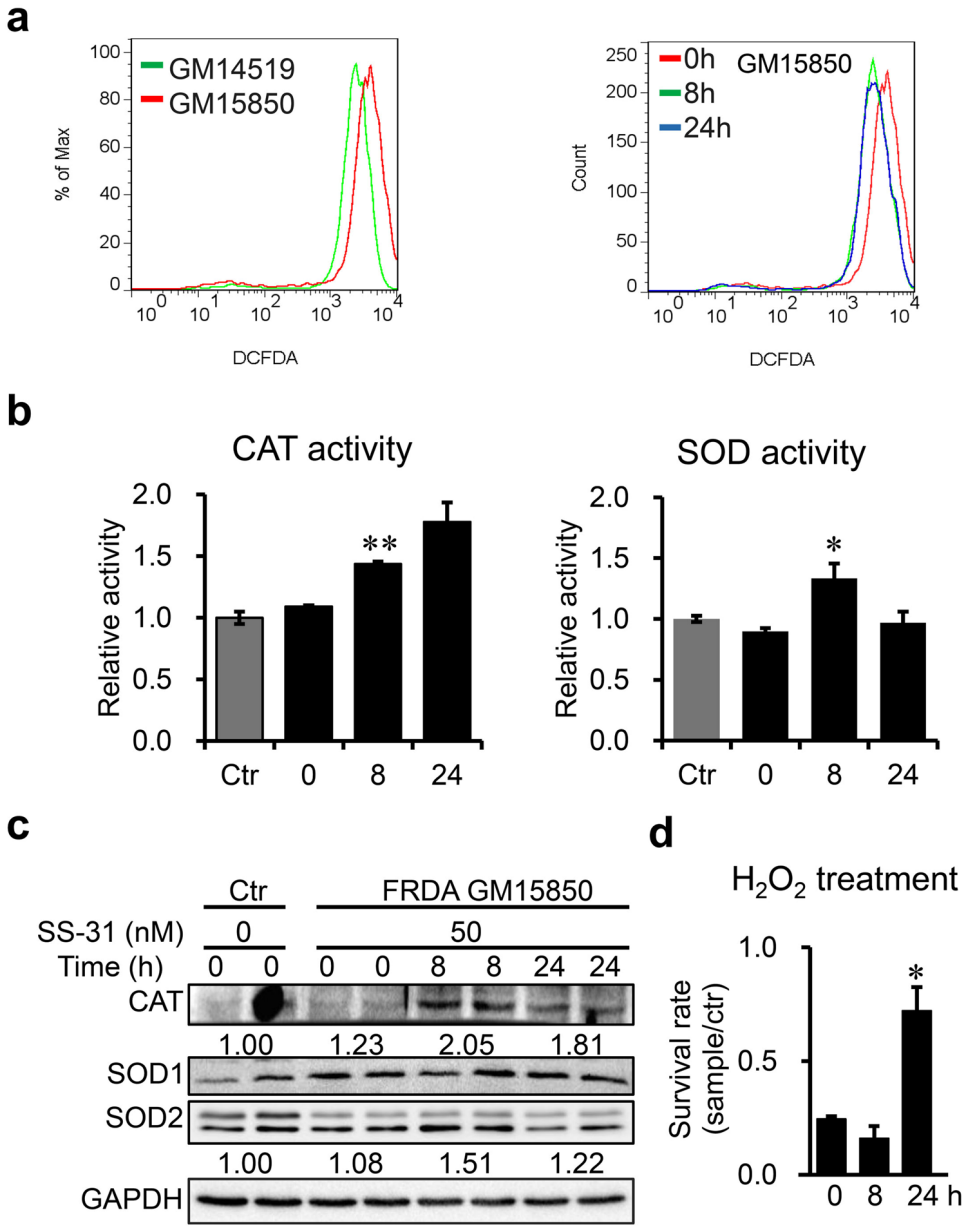
SS-31 treatment enhances the patient cells (GM15850) to defend against oxidative stress. (**a**) Level of the cellular ROS detected with probe DCFDA by Flow cytometry. Left panel for control cells (GM14519) and patient derived cells (GM15850); right panel for GM15850 cells after SS-31 treatment for 8 or 24 h. (**b**) Enzymatic activities of catalase (CAT) and superoxide dismutase (SOD). (**c**) Representative graphs for protein levels CAT and SOD1 and 2, revealed by Western blotting. (**d**) Cell survival rate of GM15850 after SS-31 treatment followed by challenge with H_2_O_2_. GM15850 cells were treated with 50 nM SS-31 for 8 or 24 h, then 100 µM H_2_O_2_ challenge for 1 h. After wash once with culture medium, CCK8 was used to determine the survival rate. Ctr: +SS-31−H_2_O_2_; sample: +SS-31+H_2_O_2_. Values represent mean ± SEM (n = 3, each duplicates). A one-way ANOVA was performed, *p < 0.05, **p < 0.01 compared with the rate at time point 0.

An increasing amount of data from different organisms support the notion that frataxin-deficiency results in oxidative stress, which is at least one of the pathological mechanisms of FRDA^30^. Reduction of oxidative stress by preventing ROS generation and/or enhancing the ROS scavenging ability may be a promising target for therapy^31^. To estimate the effect of SS-31 on the level of oxidative stress in patient-derived cells, we used the ROS probe DCFH-DA to analyse cellular ROS production by flow cytometry. The result showed that ROS levels were higher in the patient-derived lymphoblasts than in healthy control cells (Fig. 5a, left panel). SS-31 treatment drastically decreased ROS levels in patient-derived lymphoblasts (Fig. 5a, right panel). To reveal if the reduction in ROS levels stemmed from the ROS scavenging ability, we detected the protein levels and enzymatic activities of superoxide dismutase (SOD) and catalase, which are important cellular scavengers of superoxide and hydrogen peroxide (H_2_O_2_), respectively. SS-31 treatment increased the expression and activity of catalase (Fig. 5b, left panel and c). Although SS-31 treatment did not change the protein level of SOD1 and SOD2 in patient-derived cells, total SOD activities significantly increased after incubation for 8 h with SS-31 (Fig. 5b, right panel and c). We then examined if patient-derived cells became more resistant to oxidative stress challenge after SS-31 treatment. Cell survival was estimated using a CCK8 viability assay after exposure to H_2_O_2_. Intriguingly, patient-derived lymphoblasts showed significantly more resistance to H_2_O_2_-mediated cellular toxicity after SS-31 treatment than before treatment (Fig. 5d). Altogether, these data suggest that SS-31 treatment diminishes ROS levels and enhances anti-oxidative ability of cells derived from FRDA patients.

### SS-31 translationally upregulates FXN expression

**Figure 6:**
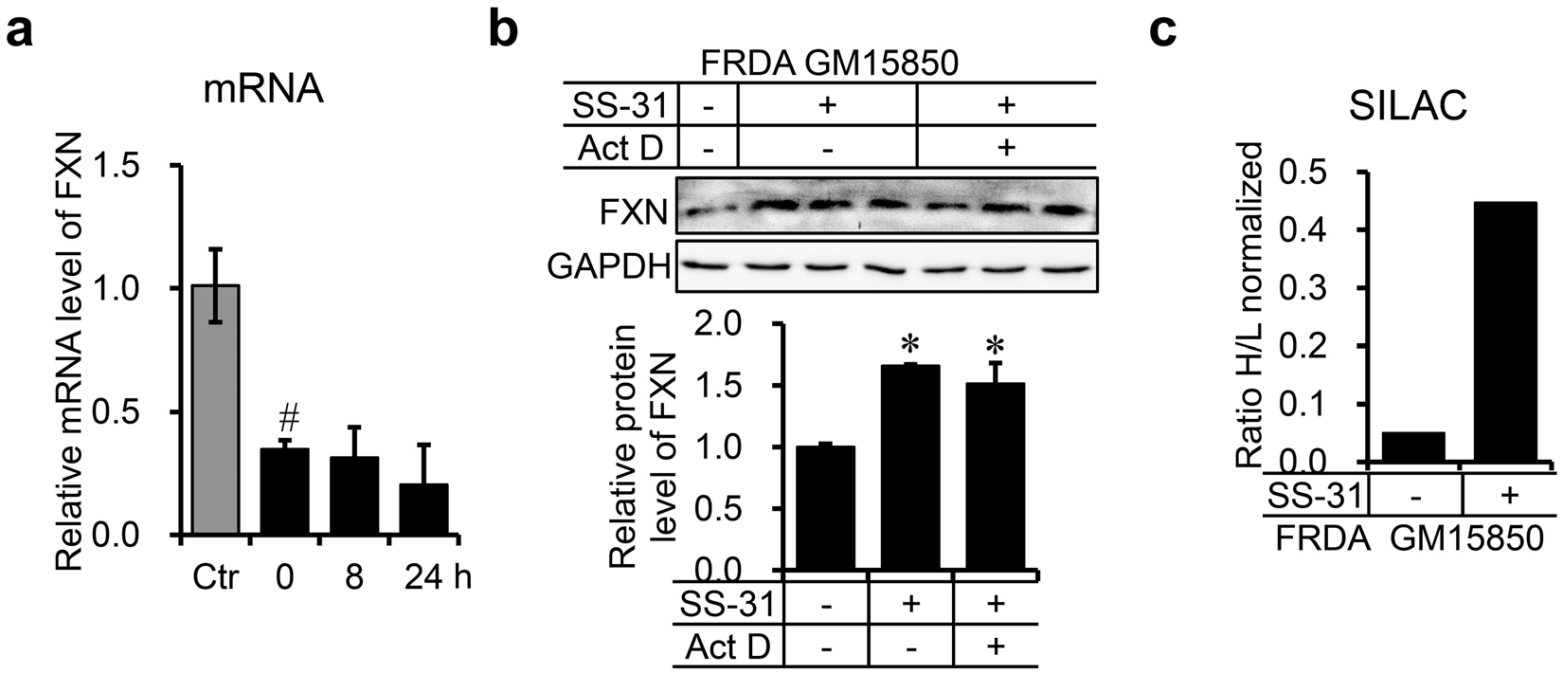
SS-31 treatment translationally upregulates FXN expression. (**a**) mRNA level of *FXN* in GM15850 cells after treatment with 50 nM of SS-31 for 8 or 24 h. Total RNA was extracted from the cells after treatment and analysed by qRT-PCR. The relative level was normalised to that in healthy GM15849 cells. *GAPDH* was used as an internal control. Values represent mean ± SEM (n = 4, each duplicates). (**b**) Protein level of FXN in GM15850 cells after co-treatment with 50 nM of SS-31 and 0.5 µg/mL of actinomycin D (ActD). The cells were collected after 8 h and the FXN protein level was detected by Western blotting. Upper panel: a representative graph of western results; lower panel: quantitative data of the intensity of the western bands. Values represent mean ± SEM (n = 3, each duplicates). A one-way ANOVA was carried out, ^#^ *p* < 0.05 compared with the healthy control GM14519 cells. *p < 0.05 compared with the untreated GM15850 cells. (**c**) Ratio of newly synthesised FXN after SS-31 treatment to before-treatment synthesised protein revealed by SILAC technology in GM15850 cells. The Cells were cultured in conventional RPMI 1640 medium containing ^12^C^14^N-Lysine and ^12^C^14^N-Arginine (“light”, L) overnight, then transferred into medium containing ^13^C_6_^15^N_2_-Lysine and ^13^C_6_^15^N_4_-Arginine (“heavy”, H) for 24 h with/without 50 nM of SS-31 (see Materials and Methods). Immunoprecipitation was performed with FXN antibody to enrich total FXN protein, which was sent for SILAC assay. No significance data for (**c**) due to only two repeats.

To investigate how SS-31 increases the protein level of FXN, we measured both the mRNA and protein levels of FXN. Treatment with SS-31 for 8 h resulted in no increase in the mRNA level of FXN (Fig. 6a), but a marked increase in FXN protein levels (Fig. 1), suggesting posttranscriptional regulation of FXN expression. To confirm this, we treated the cells with SS-31 and simultaneously with actinomycin D to block gene transcription. Protein levels of FXN were then determined with immunoblotting. The result showed that the protein level of FXN was still elevated after 8 h treatment (Fig. 6b), further supporting translational or post-transcriptional upregulation. To further confirm that the increased FXN was indeed newly synthesised FXN, we used the SILAC (Stable isotope labelling with amino acids in cell culture) technology to distinguish newly synthesised FXN after SS-31 treatment from the FXN synthesised before SS-31 treatment. Figure 6c clearly showed that translation was markedly enhanced by SS-31 treatment. Combined with our previous study in which we showed that the half-life of FXN is about 48 h^21^, the current results demonstrate that SS-31 treatment translationally upregulates FXN expression, pointing to its potential in the treatment of FRDA.

## Discussion

**Figure 7:**
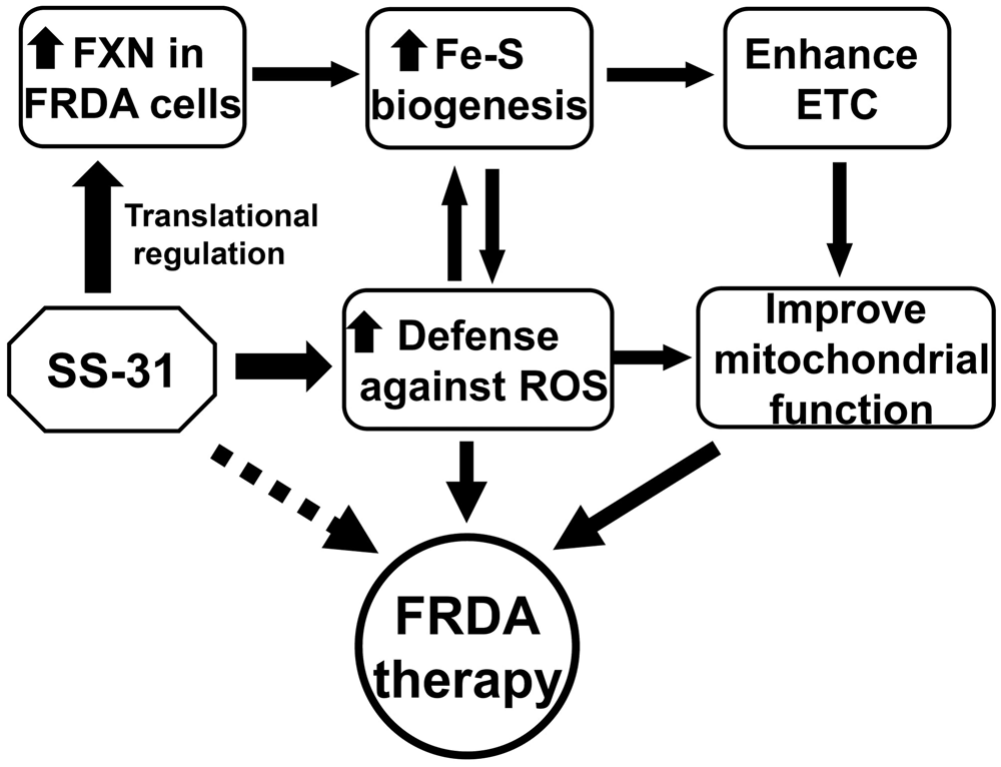
A working model of SS-31 efficacy in FRDA patient-derived cells in this study. On the one hand, SS-31 increases FXN expression and available iron in mitochondria, reverses the deficiency of iron-sulphur cluster biogenesis and enhances the ETC of respiratory chain and ATP production. On the other hand, SS-31 reduces the ROS level and increases the capacity for defence against oxidative stress. Altogether, SS-31 improves the mitochondrial function of FRDA patient-derived cells through two pathways and acts as a potential drug for the treatment of FRDA.

Currently no approved therapy is available for FRDA, and only limited treatment for the management of symptoms is available to patients^1, 32^. Since the lack of mitochondrial FXN is the root cause, increasing the amount of FXN will be the most effective therapeutic approach^22^. The target cell types in FRDA, cardiocytes and neurons, are difficult to study *in vitro*. In contrast, lymphocytes from FRDA patients are more straightforward to study due to the very low level of FXN protein compared with healthy control cells. This lymphoblast model has been widely used in the FRDA field^33–35^. In this study, we demonstrate that SS-31 not only acts as an effective antioxidant, but also, most interestingly, rescues FXN deficiency in FRDA patient-derived cells. SS-31-induced upregulation of FXN occurs translationally and in a dose-dependent manner. Promisingly, enzymatic activities of most of the iron-sulphur cluster-containing enzymes tested increased and the quality of mitochondria was greatly improved after SS-31 treatment. In addition, SS-31 as an antioxidant not only reduced ROS accumulation, but also boosted the cell’s ability to defend against oxidative stress. Therefore, the beneficial effect of SS-31 on the lymphoblasts derived from FRDA patients could result from these two mechanisms as described and modelled in Fig. 7.

Many studies showed that SS-31 is effective in cellular and animal models for a few diseases that are associated with bioenergetic failure^36, 37^. Due to the superior properties of SS-31, more than 20 clinical trials (registered as elamipretide or Bendavia) have been being conducted in phase 1 or 2 (https://clinicaltrials.gov/), covering a number of mitochondrial disorders. So far, the safety and tolerability of SS-31 were well confirmed^38^. The efficacy of SS-31 revealed in the current study raises it as a candidate for a therapeutic strategy of FRDA treatment.

A deficit in iron-sulphur cluster biogenesis is considered the primary effect of FXN deficiency, a notion supported by a number of studies showing that the activities of the iron-sulphur cluster-dependent aconitase and mitochondrial respiratory chain complexes I, II and III drastically reduce in tissues of FRDA patients^8, 39, 40^. Here, SS-31 treatment induced upregulation of FXN and elevated the activities of iron-sulphur enzymes, such as mitochondrial aconitase and complex II and III, but not XOD and complex I. XOD is an enzyme that needs both iron-sulphur cluster and molybdenum cofactor as prosthetic groups to be functional, to generate cytosol-derived ROS. The lack of increased enzymatic activity of XOD after SS-31 treatment suggests iron-sulphur cluster alone is not enough to promote the activity of XOD. Complex I activity was not recovered, possibly because much more iron sulphur clusters are needed for complex I than other enzymes. Complex I is also the biggest complex with 46 subunits, which may take more time to be expressed and finely assembled than other complexes. Therefore, longer administration of SS-31 may be needed in clinical trials to reach full-scale beneficial effects.

Early histological and biochemical studies into the pathophysiology in patient samples revealed that dysregulation of iron metabolism is a key feature of the disease, mainly characterised by mitochondrial iron accumulation and cytoplasmic iron deficiency. Consistent with our previous study^21^, we found that IRP2 is upregulated and ferritin is downregulated in FRDA patient derived cells. After SS-31 treatment, the protein level of FXN increased along with ferritin upregulation, indicating more iron uptake into cytosol for ferritin to consume. Therefore, ferritin can be stabilised. Meanwhile, absorbed iron may bind the iron responsive element to activate the translation of ferritin^41^. Unexpectedly, IRP2 expression was still elevated, indicating iron-demand signalling. To track the absorbed iron after SS-31 treatment, we measured cytosolic and mitochondrial LIP levels with Calcein-AM and RPA, respectively. We found an increase in mitochondrial LIP level, but no changes in cytosolic LIP level after SS-31 treatment. Notably, no significant alteration in the concentration of mitochondrial LIP iron could be observed in cultured patient-derived cells, which had also been observed previously^42^. The results suggest that newly taken-up iron is supplied to ferritin and/or directly goes into mitochondria as bioavailable iron for iron-sulphur cluster biogenesis.

Oxidative damage and mitochondrial dysfunction are the key determinants of cellular damage in FRDA patient cells^43^. FXN deficient cells are much more sensitive to oxidative stress than wild type cells^9^. It has been shown that cells with FXN deficiency also have an impaired ability to recruit antioxidant to defend against endogenous oxidative stress^9, 44^. Several therapies are aimed at reducing the consequences of FXN deficiency, including antioxidants to reduce cellular oxidative damage, such as idebenone, MitoQ, CoQ10, and vitamin E. However, clinical trials neither support nor refute an effect of antioxidants (idebenone, or a combination of coenzyme Q10 and vitamin E) so far on the neurological status of people with FRDA^45^. Many studies have shown that SS-31 can scavenge various ROS in animal models of neurodegenerative diseases^46, 47^. In our cell model, SS-31 drastically decreased ROS level in patient-derived cells (Fig. 5a). Furthermore, the cells after SS-31 treatment exhibited significantly more resistance to H_2_O_2_-mediated cytotoxicity than the cells before treatment by increasing the enzymatic activities of SOD and catalase (Fig. 5b), two important ROS scavengers. Therefore, we conclude that SS-31 treatment reduces the ROS generation and enhances the competence to scavenge the ROS.

To judge the therapeutic strategy for a mitochondrial disease, any cellular improvement after drug treatment needs to be checked through mitochondrial morphology and function. SS-31 can improve the mitochondrial function in a number of cellular and preclinical models^48, 49^. Our results demonstrate that SS-31 is very effective in restoring the MMP, increasing the ratio of NAD^+^/NADH, and promoting ATP production in FRDA patient-derived cells. Even mtDNA copy number mildly increased after SS-31 treatment, suggesting restored function. Though it was reported that SS-31reduced fission and increased fusion activity in mutant Htt neurons^50^, we did not find any clear difference when counting mitochondrial numbers (not shown). Remarkably, mitochondrial morphology changed structurally from disorganised into regular, finely arrayed cristae. However, SS-31 had no effect on mitochondrial morphology, mitochondrial proteome, respiration, ATP synthesis or ROS production in young healthy animals^26, 51, 52^. All the melioration in patient-derived cells suggests that SS-31 improves mitochondrial quality.

In summary, SS-31 treatment improves the morphology and function of mitochondria in the FRDA patient-derived cells by upregulating the expression of FXN at the translational level and reducing oxidative stress. In addition, SS-31 treatment significantly enhances the ability of patient-derived cells to withstand challenges from exogenous oxidative stress. The mechanism of the translational upregulation in FXN expression by mitochondrion-targeted SS-31 needs to be further addressed. However, improvement in the quality of mitochondria in FRDA patient–derived cells by SS-31 treatment appears promising. It is reasonable to suggest that SS-31 might potentially be a new drug for the early treatment of FRDA.

## Materials and Methods

### Cell culture

Human healthy control and FRDA patient-derived lymphoblasts and fibroblasts were purchased from the Coriell Institute for Medical Research repository. The cell lines used: GM14519, GM15849, GM15850, MCH46, GM03816, GM04078. Cells were cultured in medium RPMI 1640 (Solarbio Science & Technology Co. Ltd., Beijing, China) with 10% FBS, penicillin (100 U/mL) and streptomycin (100 mg/mL) at 37 °C in a 5% CO_2_ tissue culture incubator. SS-31 (synthesised by ChinaPeptides Co., Ltd, Shanghai, China) was dissolved with PBS and stored in −80 °C.

### Western blot analysis

Cells were first rinsed in ice-cold phosphate-buffered saline (PBS), pH7.2, and lysed in 1% NP-40, 125 mM Tris, pH6.8, containing a protease inhibitor cocktail (Roche, Basel, Switzerland). For western blotting, 35–50 µg protein was added per lane of 12% SDS-PAGE. Primary antibodies were diluted in primary antibody dilution buffer (Beyotime Biotech., Shanghai, China). Antibodies used included: anti-GAPDH (Abgent Biotech. Co. Ltd., Suzhou, China), anti-ferritin, SOD1, SOD2, SDHB and ISCU (Abcam, Cambridge, MA, USA), anti-catalase (Santa Cruz Biotech., Santa Cruz, CA, USA), anti-XOD antibody (ProteinTech Inc., Chicago, IN, USA), anti-TfR1 antibody (Zymed, San Francisco, CA, USA), anti-FXN, IRP1, and IRP2 (polyclonal, raised from rabbit). Detection was performed using peroxidase-conjugated secondary antibodies (Thermo Fisher Scientific, Waltham, UK). Quantification of the density of the western bands was done with programme ImageJ (http://rsb.info.nih.gov/ij/).

### Labile iron pool detection

Labile iron was measured using the iron-sensitive probes Calcein-AM (Aladdin, Shanghai, China) and RPA (Squarix GmbH, Germany), for cytosolic and mitochondrial compartments, respectively. Briefly, 2 × 10^5^ cells were incubated with 10 µM Calcein-AM or RPA at 37 °C for 15 min. After two cycle washes with HBSS, cells were obtained in 100 µL HBSS or PBS. Fluorescence was revealed using the fluorescence microplate reader at 485 nm (excitation), 530 nm (emission) for Calcein-AM and 530 nm (excitation) and 601 nm (emission) for RPA.

### Measurement of the enzymatic activities of aconitase and complex I, II, and III

In-gel aconitase activity assays were performed as described previously^53^. The NADH dehydrogenase activity of isolated mitochondria was measured using the complex I enzyme activity microplate assay kit (Abcam). The procedure was adapted from the previous report^54^. The complex II assay was performed with the Succinate-coenzyme Q reductase activity assay kit (Comin Biotechnology Co., Suzhou, China) following the manufacturer’s instructions. The activity was calculated by measuring the reduction of the absorbance of 2,6-dichloroindole at 605 nm. The Mitochondrial complex III activity was measured, based on the reduction of cytochrome c through the activity of complex III following the manufacturer’s instructions (Biovision Inc., Milpitas, CA, USA).

### Determination of mitochondrial membrane potential (MMP) level and ATP content

MMP level was determined using a 5,5′,6,6′-tetrachloro-1,1′,3,3′ tetraethylbenzimidazolylcarbocyanine iodide (JC-1) mitochondrial membrane potential detection kit (Solarbio Science & Technology Co. Ltd.). The fluorescent intensity (excitation 490 nm, emission 520 nm) was determined using a fluorescence microplate reader. ATP levels were measured in cells derived from healthy control and FRDA patients by ATP content assay kit (Beyotime Biotech). The bioluminescence assay is based on the reaction of ATP with recombinant firefly luciferase and its substrate luciferin. Luciferase catalyses the formation of light from ATP and luciferin, which was measured using a luminometer.

### Measurement of cellular ROS production and SOD, catalase, and XOD activity

Intracellular ROS levels were estimated using a fluorescence-labelled probe DCFH-DA (Beyotime Biotech.) with flow cytometry analysis. Cellular SOD, catalase, XOD activities were measured following the manufacturer’s instructions (Nanjing Jiancheng Bioengineering Institute, Nanjing, China).

### Cell viability assays

Cells were treated with 50 nM of SS-31 for 8 or 24 h, then transferred (10000 cells/100 ul/well) into 96-well flat-bottom tissue culture plates. Cultures were supplemented with 100 µM H_2_O_2_. After an hour, the reagent (Cell Counting Kit-8, Dojindo, Kumamoto, Japan) was added (10 μL/well) and cells were incubated for additional 4 h. Results were expressed as the percentage of reduction of absorbance at 450 nm by calibration with the absorbance of the control (untreated) cells.

### Quantitative real-time PCR

Total genomic DNA was extracted from the cells using Genomic DNA Mini Preparation Kit with Spin Column (Beyotime Biotech.) and eluted in sterile distilled H_2_O. Total RNA was extracted using TRizol (Bioteke, Beijing, China), and converted to cDNA with Transcriptor First Strand cDNA Synthesis Kit (Roche). Total genomic DNA or cDNA was subjected to qPCR analysis with SYBR Green Master Mix (Thermo Fisher Scientific) in Applied Biosystems 7300. Results were considered positive when the fluorescent signal above the baseline was detected and the cycle number was recorded. The relative copy number of a gene was determined by calibration with the internal control (*GAPDH* for RNA, *β-actin* for DNA). Primers for the genes are listed as follows (gene: forward primer; reserve primer):

*FXN*: 5′-CCTTGCAGACAAGCCATACA-3′; 5′-CCACTGGATGGAGAAGATAG-3′; *GAPDH*: 5′-TGCACCACCAACTGCTTAGC-3′; 5′-GGCATGGACTGTGGTCATGAG-3′; *CytB*: 5′-TATCCGCCATCCCATACATT-3′; 5′-GGTGATTCCTAGGGGGTTGT-3′; *β-actin*: 5′-AGAAAATCTGGCACCACACC-3′; 5′-AACGGCAGAAGAGAGAACCA-3′

### Stable isotope labelling with amino acids in cell culture (SILAC)

SILAC is a simple and straightforward approach for *in vivo* incorporation of a label into proteins for mass spectrometry (MS)-based quantitative proteomics. Cells were cultured in conventional RPMI 1640 medium containing ^12^C^14^N-Lysine and ^12^C^14^N-Arginine (“light”) overnight. The cells were then transferred into RMPI 1640 medium containing ^13^C_6_^15^N_2_-Lysine and ^13^C_6_^15^N_4_-Arginine (“heavy”) (Sigma) with or without 50 nM of SS-31. After 24 h, the cells were collected and lysed with lysis buffer. Total FXN was enriched by immunoprecipitation with FXN antibody and assayed by LC-MS/MS using machine Q Exactive (Thermo Fisher Scientific). Newly synthesised FXN containing the “heavy” isotopes is distinguishable from the “old” FXN containing the “light” isotopes.

### Statistics analysis

Data were presented as mean ± SEM from 3 independent experiments. A one-way analysis of variance (ANOVA) was done using SPSS ver. 22.0 software (IBM Corporation, Armonk, NY, USA). Significance was considered at *p* < 0.05.

## Electronic supplementary material

Supplementary Information
