# Supplementary material for: Peptide SS-31 upregulates frataxin expression and improves the quality of mitochondria: implications in the treatment of Friedreich ataxia
Source: Sci Rep. 2017 Aug 29;7:9840. doi: 10.1038/s41598-017-10320-2 (PMC5575096; doi:10.1038/s41598-017-10320-2)
Supplement: Supplementary file 1 — Supplementary Information [file 41598_2017_10320_MOESM1_ESM.pdf]

Peptide SS-31 upregulates frataxin expression and improves the quality of mitochondria: implications in the treatment of Friedreich ataxia

Hongting Zhao[1], Huihui Li[1], Shuangying Hao[1,2], Jiping Chen[1], Jing Wu[1], Chuanhui Song[1], Meng Zhang[3], Tong Qiao[3], Kuanyu Li[1]*

[1]Jiangsu Key Laboratory of Molecular Medicine, Medical School of Nanjing University, 210093 Nanjing, P. R. China

[2]Current Address: Medical School of Henan Polytechnic University, 454000 Jiaozuo, P. R. China

[3]Department of Vascular Surgery, Drum Tower Clinical Medical College of Nanjing Medical University, Nanjing, P. R. China

*To whom correspondence should be addressed:

Tel: +86-25-8359-4765;

Fax: +86-25-8368-6550;

E-mail: likuanyu@nju.edu.cn

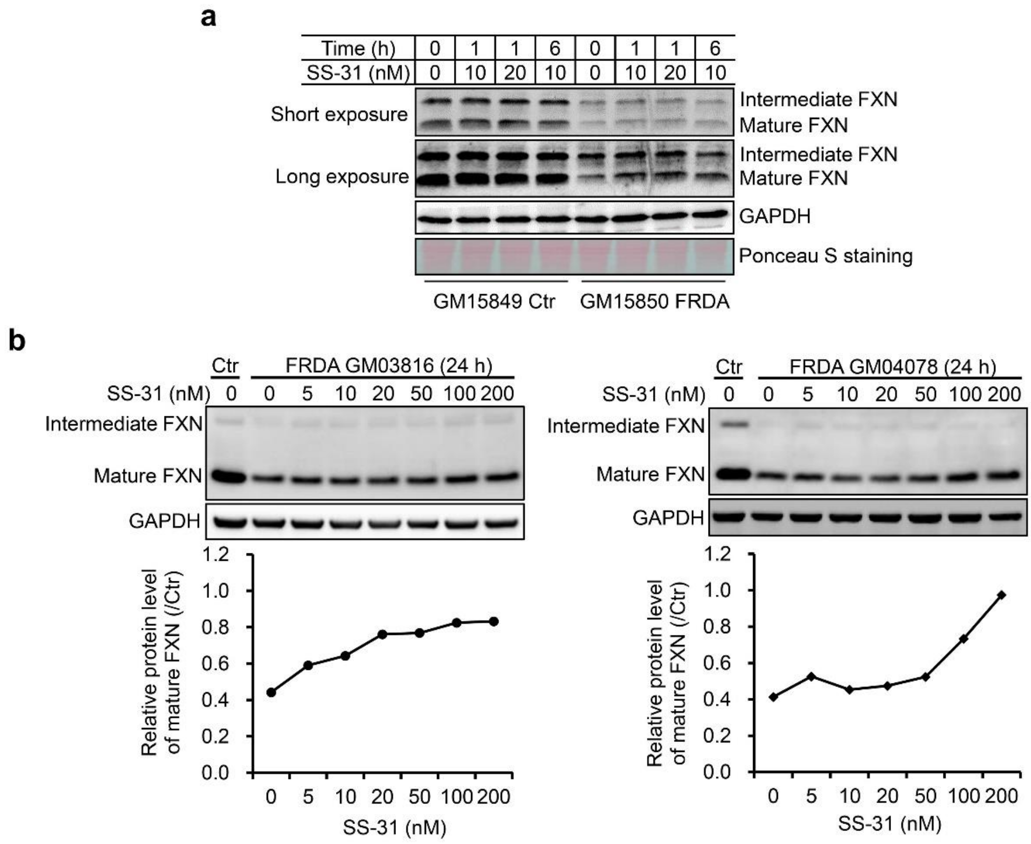

Figure S1. SS-31 upregulates FXN expression in lymphoblasts and fibroblasts derived from FRDA patients, but not in GM15849 cells derived from healthy control. (a) Western Blotting analysis for protein level of FXN in healthy control cells (GM15849) and patient derived cells (GM15850) after treatment with SS-31 in different concentrations and time points. (b) Western Blotting analysis for protein level of FXN in fibroblasts GM03816 and GM04078 derived from FRDA patients after treatment with SS-31 in different concentrations.

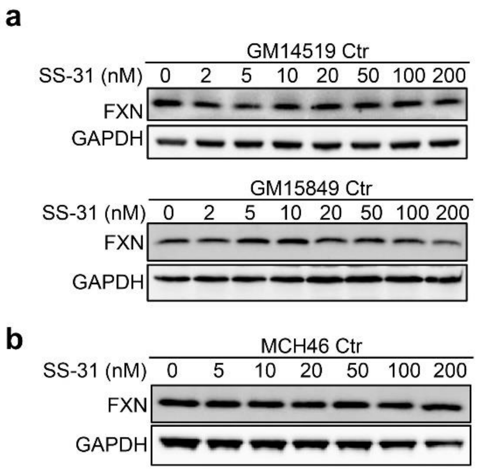

Figure S2. SS-31 has no effect on FXN expression in lymphoblasts (GM15819 and GM15849, **a**) and fibroblasts (MCH46, **b**) derived from healthy controls. Cells were collected for Western blotting 24 h post SS-31 treatment within a broad range of concentration, same as the treatment for GM15850 in the manuscript.

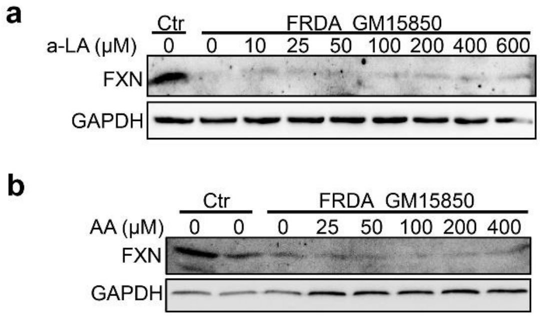

Figure S3. Neither alpha-lipoic acid (α-LA) nor ascorbic acid (AA) treatment has effect on the protein level of FXN in FRDA patient-derived cells (GM15850). Protein levels of FXN were shown in GM15850 cells after treatment with α-LA or AA in different concentrations for 24 h.

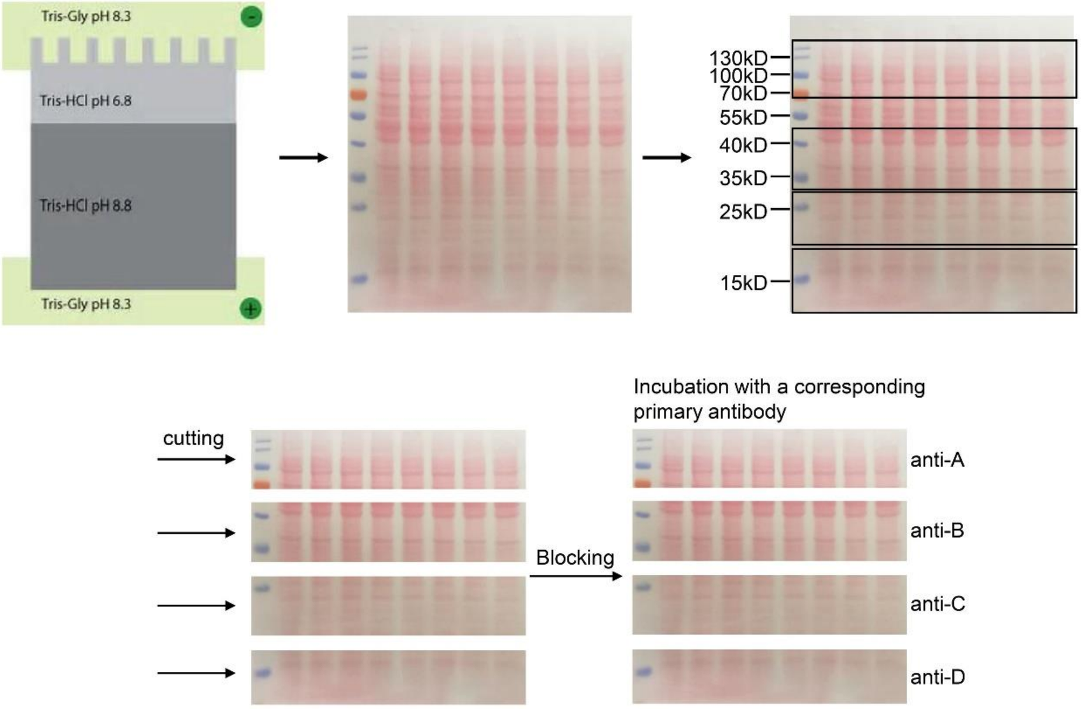

Figure S4. This is the schematic diagram for blots cut from a same entire blot into small pieces as needs. The main steps include 1. Gel running; 2. Blotting and Ponceau S staining; 3. Determining the areas to cut as needs; 4. Cutting into different pieces; 5. Blocking; 6. Antibody incubation for determination of protein levels. In case the molecular weight of a protein is similar to another, then the second or even the third gel is run with the same samples. The following results were got from this strategy. Here we show the representative figures presented in the manuscript.

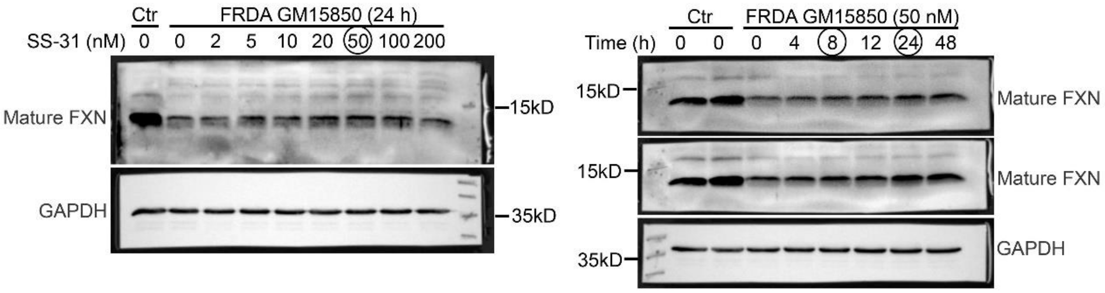

Figure S5. This is the raw data of Fig.1a and b. The grouping of blots is from different parts of a same gel.

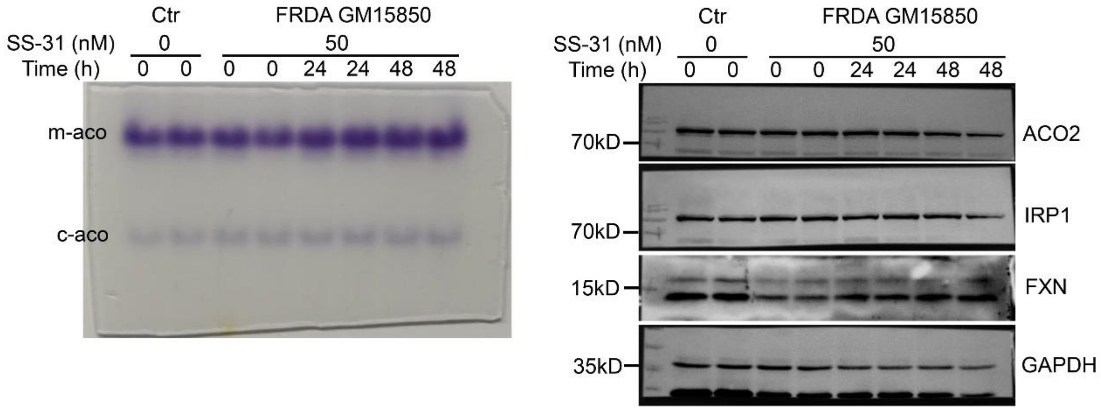

Figure S6. This is the raw data of Fig.3a. The left is a representative figure for aconitase activities from an in-gel assay. The right panel is a representative figure for Western analysis from the same samples as in the left panel.

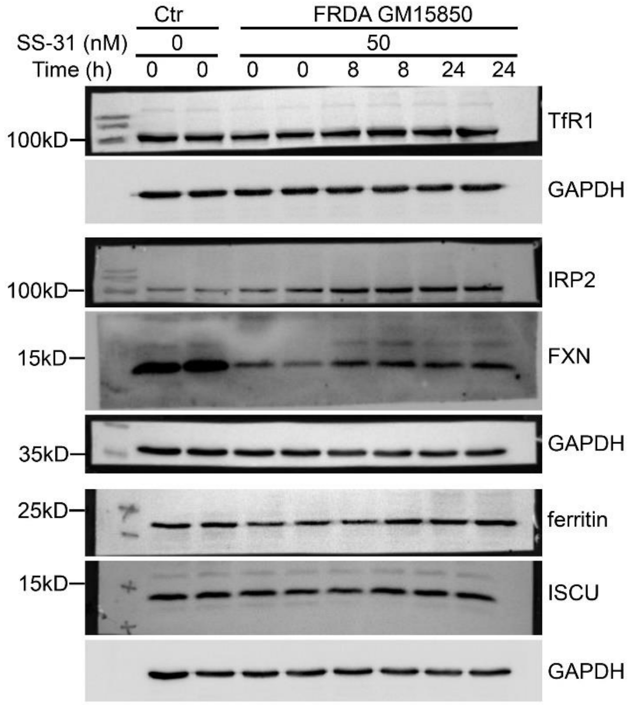

Figure S7. This is the raw data of Fig.4a. The grouping of blots is from three gels but with the same samples, e.g. TfR1 and the corresponding internal control GAPDH from the first gel, IRP2, FXN, and the corresponding GAPDH from the second gel, ISCU, ferritin, and their internal control GAPDH from the third gel.

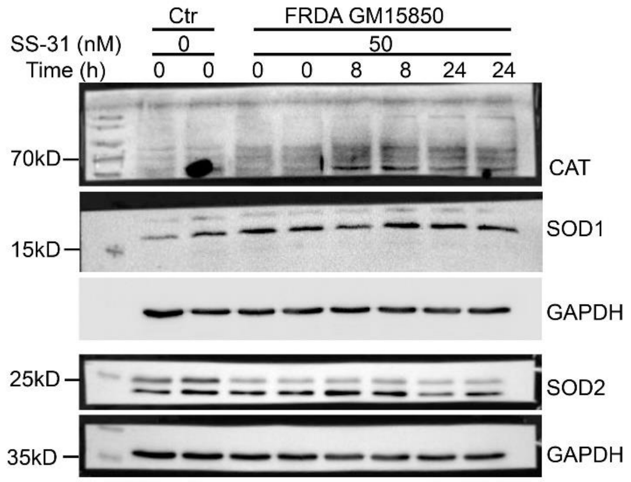

Figure S8. This is the raw data of Fig.5c. The grouping of blots is from different gels, CAT, SOD1, and their internal control GAPDH from the first gel, SOD2 and its internal control GAPDH from the second gel.

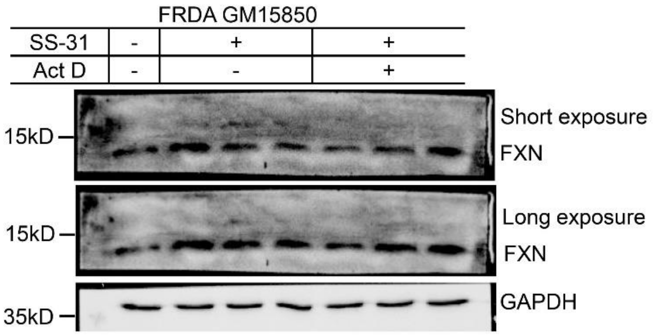

Figure S9. This is the raw data of Fig.6b. The grouping of blots is from a same gel.
